# Association of Physical Activity With Risk of Mortality Among Breast Cancer Survivors

**DOI:** 10.1001/jamanetworkopen.2022.42660

**Published:** 2022-11-17

**Authors:** Lie Hong Chen, Michael R. Irwin, Richard Olmstead, Reina Haque

**Affiliations:** 1 Department of Research & Evaluation, Kaiser Permanente Southern California, Pasadena, California; 2 Department of Health Systems Science, Kaiser Permanente School of Medicine, Pasadena, California; 3 Cousins Center for Psychoneuroimmunology, Semel Institute for Neuroscience and Human Behavior, UCLA Geffen School of Medicine, Los Angeles, California

## Abstract

This cohort study evaluates the association of physical activity with risk of all-cause mortality among active and moderately active breast cancer survivors.

## Introduction

The protective effect of physical activity on risk of developing breast cancer is known; however, its effect after breast cancer diagnosis remains controversial. It has been suggested that both moderate and strenuous exercise have comparable benefits,^1,2^ but survival outcomes have been studied rarely among patients with cancer. The aim of this study was to evaluate the association of physical activity beyond essential daily functioning with risk of all-cause mortality among breast cancer survivors.

## Methods

We conducted a cohort study of postmenopausal breast cancer survivors who had received their initial diagnosis at least 2 years previously (median 6 years of survivorship) and were members of a health care plan in California. Participants were initially diagnosed with early-stage breast cancer (American Joint Committee on Cancer TNM stages 0-II) between 1996 and 2012. Baseline interviews were conducted between August 1, 2013, and March 31, 2015, and participants were followed up until date of death or study’s end (April 30, 2022). Participants were queried about leisure time physical activity and fatigue using 2 validated questionnaires, the Godin-Shephard Leisure-Time Physical Activity Questionnaire (GSLTPAQ)^3^ and the Fatigue Severity Inventory.^4^ The GSLTPAQ assessed exercise during a typical 7-day period of at least 15 minutes’ duration and provided a composite score that categorized exercise patterns as active, moderately active, or insufficiently active at baseline (eMethods in Supplement 1). Death dates were ascertained from the health plan and from state and national death databases. A Cox proportional hazards regression model was used to estimate the association of physical activity with risk of all-cause mortality, adjusted for age at baseline, breast cancer stage, fatigue, Charlson Comorbidity Index, years since cancer diagnosis, self-reported race and ethnicity (included because breast cancer survivorship studies have associated race and ethnicity with the exposure variable [ie, activity level] and outcome [ie, death]), lifetime history of insomnia and depression, and adjuvant cancer treatments (endocrine, chemotherapy and radiation). Statistical analyses were performed with SAS, version 9.4 (SAS Institute Inc), and a 2-sided *P* < .05 was considered statistically significant.

The study protocol was reviewed and approved by the Kaiser Permanente Southern California Institutional Review Board, and all participants provided informed consent. We followed the STROBE reporting guideline.

## Results

The study included 315 participants, all of whom were women. The mean age at interview was 71 years (range, 57-86 years); 66 participants (20.9%) were African American or Black, 28 (8.9%) were Asian or Pacific Islander, 4 (1.3%) were Hispanic, and 217 (68.9%) were non-Hispanic White. Over a maximum follow-up of 8.7 years (median, 7.8 years [IQR, 7.3-8.3 years]) after baseline, 45 participants (14.3%) died due to any cause, 5 of whom died due to breast cancer. The mortality rates were 12.9/1000 person-years (PY) for active participants, 13.4/1000 PY for moderately active participants, and 32.9/1000 PY for insufficiently active participants. On multivariable analysis, compared with insufficiently active participants, those who were active or moderately active had a 60% decreased risk of death (active: hazard ratio (HR), 0.42 [95% CI, 0.21-0.85]; moderately active: HR, 0.40 [95% CI, 0.17-0.95]) (Table), consistent with the Kaplan-Meier curves (Figure). Treating GSLTPAQ score (baseline up to 32 months) as a time-dependent variable produced a similar association.

**Table.** Multivariable Adjusted Risk of All-Cause Mortality by GSLTPAQ Scores^a^

Variable	No. of participants	No. of deaths	PY	Rate per 1000 PY (95% CI)	Adjusted HR (95% CI)
Total	315	45	2382.3	18.9 (14.1-25.3)	NA
GSLTPAQ score^b^					
Active (≥24 units)	141	14	1086.4	12.9 (7.6-21.8)	0.42 (0.21-0.85)
Moderately active (14-23 units)	77	8	596.5	13.4 (6.7-26.8)	0.40 (0.17-0.95)
Insufficiently active (<14 units)	97	23	699.4	32.9 (21.9-49.5)	1 [Reference]

Abbreviations: GSLTPAQ, Godin-Shephard Leisure-Time Physical Activity Questionnaire; HR, hazard ratio; NA, not applicable; PY, person-years.

^a^ Adjusted for age at baseline, breast cancer stage, fatigue, Charlson Comorbidity Index, years since cancer diagnosis, race and ethnicity, history of insomnia and depression, and adjuvant cancer treatments (endocrine therapy, chemotherapy, and radiation therapy).

^b^ Units are based on scores derived from the GSLTPAQ formula (Supplement 1).

**Figure.**
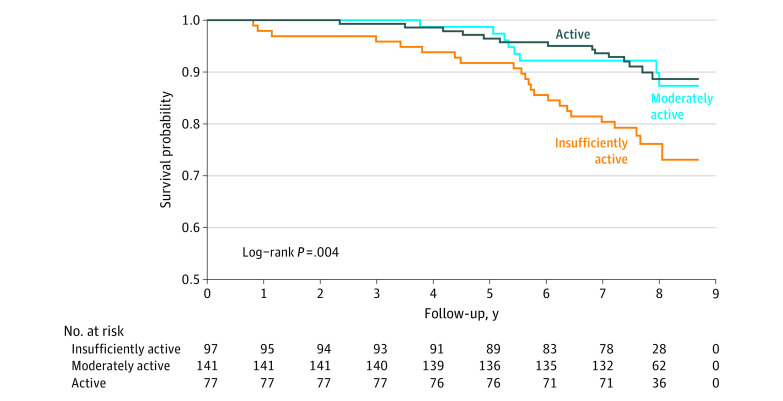
Risk of All-Cause Mortality Among Breast Cancer Survivors by Godin-Shephard Leisure-Time Physical Activity Questionnaire Scores at Baseline

## Discussion

The findings of this cohort study suggest that even moderate physical activity was associated with a 60% lower risk of death among breast cancer survivors, similar to a previous cohort.^5^ The mortality risk was similar among participants who were active and those with moderate physical activity levels. Our findings further suggest that survivorship care plans should consider incorporating physical activity because even moderate activity may be vital for extending survival as well as health-related quality of life.

The limitations of this study include a lack of information about participants’ diets and physical activity measured without the aid of technological devices. However, we assessed physical activity using validated questionnaires and accounted for several covariates, including tumor characteristics, cancer treatments, age, and comorbidity status. Our findings have implications for patient counseling on the benefits of exercise with regard to cancer outcomes,^6^ and this protection persists even after considering cancer treatments in the analysis.

## Notes

Supplement 1. **eMethods.** Godin-Shephard Leisure-Time Physical Activity Questionnaire Activity Levels

Supplement 2.
Data Sharing Statement
