# Supplementary material for: Association of Physical Activity With Risk of Mortality Among Breast Cancer Survivors
Source: JAMA Netw Open. 2022 Nov 17;5(11):e2242660. doi: 10.1001/jamanetworkopen.2022.42660 (PMC9672970; doi:10.1001/jamanetworkopen.2022.42660)
Supplement: Supplement 1. — eMethods. Godin-Shephard Leisure-Time Physical Activity Questionnaire Activity Levels [file jamanetwopen-e2242660-s001.pdf]

# Supplemental Online Content

Chen LH, Irwin MR, Olmstead R, Haque R. Association of physical activity with risk of mortality among breast cancer survivors. *JAMA Netw Open*. 2022;5(11):e2242660. doi:10.1001/jamanetworkopen.2022.42660

**eMethods.** Godin-Shephard Leisure-Time Physical Activity Questionnaire Activity Levels

This supplemental material has been provided by the authors to give readers additional information about their work.

© 2022 Chen LH et al. *JAMA Network Open.*

**eMethods.** Godin-Shephard Leisure-Time Physical Activity Questionnaire Activity Levels

Activity levels were defined using Godin leisure activity score* calculated by exercise (strenuous, moderate, and light) times per week over a 7-day period for more than 15 minutes.

| Godin scale score | Activity levels | Sample exercise |
|---|---|---|
| >24 units | Active | Strenuous or frequent exercise, e.g. running, jogging |
| 14 - 23 units | Moderate Active | Moderate exercise, e.g. fast walking, easy bicycling |
| <14 units | Insufficiently active/sedentary | Light and/or less frequent exercise, e.g. yoga, archery |

*Weekly leisure activity score = (9 x Strenuous exercise, times/week) + (5 x Moderate exercise, times/week) + (3 x Light exercise, times/week)

© 2022 Chen LH et al. *JAMA Network Open.*
