# Supplementary material for: Association of Physical Activity With Risk of Mortality Among Breast Cancer Survivors
Source: JAMA Netw Open. 2022 Nov 17;5(11):e2242660. doi: 10.1001/jamanetworkopen.2022.42660 (PMC9672970; doi:10.1001/jamanetworkopen.2022.42660)
Supplement: Supplement 2. — Data Sharing Statement [file jamanetwopen-e2242660-s002.pdf]

Data Sharing Statement

Chen. Association of Physical Activity With Risk of Mortality Among Breast Cancer Survivors. *JAMA Netw Open*. Published November 17, 2022. doi:10.1001/jamanetworkopen.2022.42660

Data
Data available: No

Additional Information
Explanation for why data not available: This dataset includes identifiers, thus investigators cannot share the dataset at this time. However, de-identified dataset may be made available to requestors following data use agreements and KPSC IRB approval. Please contact corresponding author.
